# Policy challenges for the pediatric rheumatology workforce: Part III. the international situation

**DOI:** 10.1186/1546-0096-9-26

**Published:** 2011-09-12

**Authors:** Michael Henrickson

**Affiliations:** 1 Cincinnati Children's Hospital Medical Center, 3333 Burnet Avenue, Cincinnati, OH 45229-3039, USA

**Keywords:** pediatric rheumatology, pediatric subspecialty, policy, workforce, child mortality, international health

## Abstract

Survival dominates current pediatric global health priorities. Diseases of poverty largely contribute to overall mortality in children under 5 years of age. Infectious diseases and injuries account for 75% of cause-specific mortality among children ages 5-14 years. Twenty percent of the world's population lives in extreme poverty (income below US $1.25/day). Within this population, essential services and basic needs are not met, including clean water, sanitation, adequate nutrition, shelter, access to health care, medicines and education. In this context, musculoskeletal disease comprises 0.1% of all-cause mortality in children ages 5-14 years. Worldwide morbidity from musculoskeletal disease remains generally unknown in the pediatric age group. This epidemiologic data is not routinely surveyed by international agencies, including the World Health Organization. The prevalence of pediatric rheumatic diseases based on data from developed nations is in the range of 2,500 - 3,000 cases per million children. Developing countries' needs for musculoskeletal morbidity are undergoing an epidemiologic shift to chronic conditions, as leading causes of pediatric mortality are slowly quelled.

A global crisis of health care providers and human resources stems from insufficient workforce production, inability to retain workers in areas of greatest need, distribution disparity and poor management of both health care systems and health workforce. Internationally, the pediatric rheumatology workforce will also be in very short supply for the foreseeable future relative to projected demand. Physician extenders are an essential resource to meet this demand in underserved regions. They can be trained in common aspects of musculoskeletal medicine and rheumatic conditions. Innovative strategies have been introduced in the United Kingdom to address musculoskeletal medicine educational deficiencies. Telemedicine offers an important capacity to improve access to care despite distance. Regulatory flexibility may allow realignment of clinical responsibilities through existing and future governmental or non-governmental credentialing organizations. This review explores a variety of creative approaches which hold promise to improve patient access to care.

## Review

A central mission of the pediatric rheumatology (PR) workforce is to provide children with access to care and superior clinical outcomes. This three-part series examines the many influences on workforce development, synthesizing the available data into specific policy goals. Part I detailed the unique pattern of challenges facing the PR workforce resulting from obsolete, limited or unavailable exposure to PR. Acting synergistically, the first barrier comprises three challenges. These are: a) absent or inadequate recognition or awareness of rheumatic disease by primary care providers, patients and their families; b) referral patterns that commonly foster delays in timely diagnosis; and c) primary care providers' inappropriate or outdated perception of outcomes. The second major barrier facing the PR workforce is the combined adverse effect of market competition, inadequate reimbursement and uneven institutional support. The American health care delivery system is a leading example. This barrier fosters a proliferation of varying models of PR care delivery. Generally, these versions of care delivery do not effectively improve clinical outcomes in a reliable, planned manner of longitudinal care.

Part II explored two additional national barriers and potential policy solutions for the United States (US) PR workforce. These third and fourth barriers are: 3) compromised quality of care due to current health system delivery, with limited patient access to self-management programs and multidisciplinary team care; and 4) an insufficient workforce supply available to meet the current demand. Part III examines the international challenges facing the PR workforce and the scope of available care.

The priorities in global health differ considerably from those in the US. Survival dominates global child health priorities. Cause-specific morbidity data are not reliably tracked worldwide by age. However, global mortality data are available. A brief review of the most common causes of pediatric mortality provides context for pediatric global health priorities.

For children under 5 years of age, 83-84% of global mortality can be attributed to neonatal disorders (chiefly preterm delivery, asphyxia and sepsis), pneumonia, diarrhea, malaria, measles, human immunodeficiency virus/acquired immune deficiency syndrome (HIV/AIDS), and injuries (Table 1) [1,2]. With the exception of HIV/AIDS, these seven most common causes of child (under 5 years of age) mortality are diseases of poverty. Proportional mortality varies substantially by World Health Organization (WHO) region: 74% of child deaths occur in Africa (47%) and South-East Asia (27%) [3]. The major risk factors for childhood disease and mortality are undernutrition and a lack of the fundamental social determinants of health. Undernutrition refers to calorie/macronutrient deficiency, micronutrient deficiency and the lack of exclusive breastfeeding. The most common micronutrient deficiencies are vitamin A, zinc, iron and iodine. Malnutrition encompasses overweight, obesity and undernutrition. Undernutrition alone contributes to 35% of global childhood mortality [1]. The essential social determinants of health involve poverty, inequality, lack of access to care, lack of maternal education, and exposure to conflict, war and natural disasters. Since 2008, the defined international poverty line is income below US $1.25/day based on 2005 purchasing power parity (a measure derived from relative price levels between countries) [4]. Approximately 20% of the world's population lives in this level of poverty. Children living in absolute poverty are unable to obtain basic needs (clean water, sanitation, adequate nutrition and shelter) and services (access to essential medicines, health care and education).

**Table 1:** Leading causes of mortality worldwide among children under 5 years of age [1,2]

	Cause	Proportion (%) of Total
1.	Neonatal	41
2.	Pneumonia	14
3.	Diarrhea	14
4.	Malaria	8
5.	Injuries	3
6.	HIV/AIDS	2
7.	Measles	1
8.	Other	16-17

For children 5-14 years of age, the leading causes of mortality are infectious diseases and injuries, including unintentional (traffic accidents, falls, fires, drowning and poisoning) and intentional (war, violence and self-inflicted) reasons [5]. Table 2 summarizes the leading causes in rank order for this age group (2008 data). Of the 27% of childhood deaths from injuries, 89% are consequent to unintentional and 11% from intentional causes. Musculoskeletal (MSK) diseases comprise 0.1% of pediatric all-cause mortality, of which 66% are from "other" causes (not gout, osteoarthritis or rheumatoid arthritis) and 4% from rheumatoid arthritis (by definition, not applicable to children under age 16 years). Data categorization may be problematic. Of cardiovascular disease-related deaths (3% of all-cause mortality), 0.5% are due to rheumatic heart disease.

**Table 2:** Leading cause-specific mortality worldwide among children ages 5-14 years [5]

	Cause	Proportion (%) of Total
1.	Infectious diseases	48
2.	Injuries	27
3.	Malignancies	4
4.	Cardiovascular diseases	3
5.	Neuropsychiatric conditions	3
6.	Digestive diseases	2.5
7.	Congenital anomalies	1.5
8.	Respiratory diseases	1.1
9.	Genitourinary diseases	0.9
10.	Musculoskeletal diseases	0.1
11.	Other	8-9

Overall reduction in childhood mortality and morbidity is slow and difficult to achieve [6]. Nevertheless, a global epidemiologic shift is inexorably emerging from these leading causes to chronic conditions prevalent in developed countries.

## The International Landscape

"Although the diseases that kill attract much of the public's attention, musculoskeletal conditions are the major cause of morbidity throughout the world, having a substantial influence on health and quality of life, and inflicting an enormous burden of cost on health systems [7]."

Dr. Gro Harlem Brundtland, Director General of the WHO, January 2000

**Figure 1:**
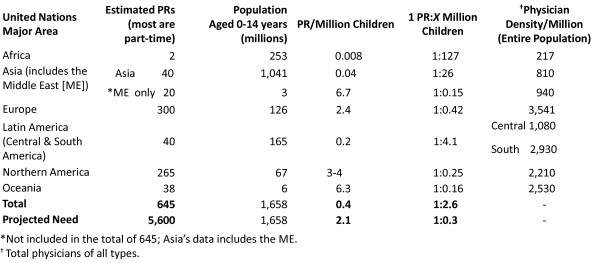
**Estimated international pediatric rheumatology workforce [11-14]**.

Using estimates of a world population of 6,809.7 million people of which 30% are children, and a range of rheumatic disease prevalence of 2,500 - 3,000 cases/1 million children [8-10], there are approximately 6-7 million children afflicted worldwide with rheumatic disease. Approximately 78% of these children live in Asia and Africa. To obtain an average acceptable density of 2.5 PRs/million children [8], the aggregate global demand requires ~5600 PRs. Figure 1 notes existing PR supply of the major areas defined by the United Nations [11-14]. In many circumstances, no data is available because there are no PRs in most developing nations. Currently, the total international PR workforce supply is 12% of this demand; the US possesses 40% of this total supply. The needs of these children are understandably eclipsed by the leading causes of pediatric mortality. However, the global epidemiologic shift to chronic conditions merits workforce development to meet the accompanying morbidity Dr. Brundtland described [15-17].

Among the 30 countries of the Organisation for Economic Co-operation and Development (OECD), only Canada, Finland, Poland, the United Kingdom (UK) and the US have formal credentialing processes for postdoctoral training in pediatric rheumatology. Non-OECD, European community member Bulgaria may also organize and certify this training [18]. Other European countries lack formalized certification of PR fellowship training. Both PR and internist rheumatologist physician resources in Canada are "inadequate" to fulfill requisite clinical care demands. Canadian surveys project a 64% shortfall in rheumatologists by 2026 [19,20].

PR has been growing rapidly in Europe. In 1999, the Pediatric Rheumatology European Society (PRES) was founded. By 2005, the UK had 180 members (including allied health professionals) in its British Society for Paediatric and Adolescent Rheumatology (BSPAR) [21]. UK trainees who seek tertiary center PR positions prepare for 2-3 years in at least two different nationally recognized centers. Those who do not seek a tertiary PR position usually receive at least a year of PR training, followed by additional pediatric training to become a consultant pediatrician with a special interest in PR. UK PRs endure frustrations with inadequate MSK medicine education at UK medical schools, delays in referral and a workforce shortage. Their educational policy approach involves promoting inclusion of pediatric MSK clinical skills and knowledge in their Competency Framework for Postgraduate General Paediatrics. This approach targets medical students with adult MSK educational tools such as the Gait, Arms, Legs, Spine (GALS) screen and its pediatric equivalent (pGALS). UK training further emphasizes medical student exposure to PR to raise awareness [22,23]. For UK physicians to whom children with MSK problems will likely present, educational research indicates their self-rated confidence in pediatric MSK assessment ranked lowest below all other bodily systems [24]. The US medical education policy approach should follow the UK PRs' lead here.

**Figure 2:**
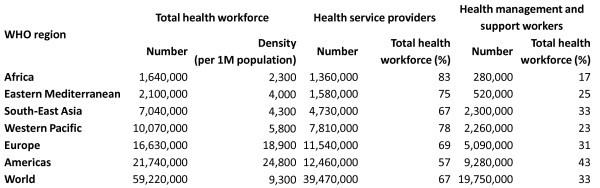
**Global health workforce, by density, 2006 [27-28]**.

**Figure 3:**
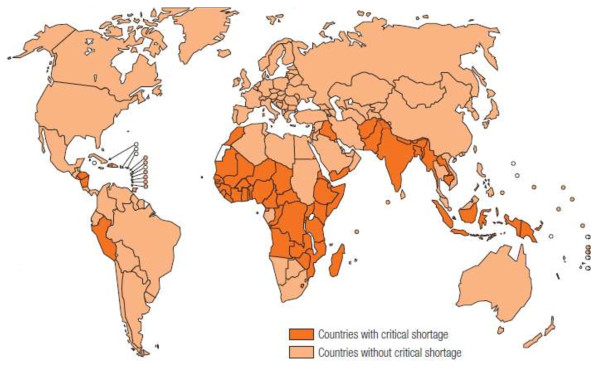
**Countries with a critical shortage of health service providers (doctors, nurses and midwives) [28]**.

The global crisis in available human resources for health will continue to limit international development of PR. The causes of this crisis are complex. They involve insufficient production, inability to retain workers in areas of greatest need, and poor management of both health care systems and health workforce. While approximately one half of the global population lives in rural locations (defined by the OECD as communities with a population density below 150 inhabitants/km^2^), these areas are served by less than a quarter of the total physician workforce [25]. The WHO recommends a minimum target density of 2.3 health workers (physicians, nurses or midwives) per 1,000 population (2,300/million). This target is a simple needs-based estimate derived by the WHO. The estimate uses the percentage of births attended by trained health workers as a proxy for: 1) health needs, and 2) the numbers of workers required to achieve 80% of births attended by a trained worker [26,27]. This recommendation does not include community and traditional health workers in specific regions or countries, e.g., the African Sahel or China, where these workers contribute extensively to routine care. Health service providers comprise two thirds of the global health workforce; the remaining third consists of health management and support workers (Figure 2) [27]. Distribution disparities exist, exemplified by the sub-Saharan African countries of Côte d'Ivoire, Mali and the Democratic Republic of Congo. These countries have a large overproduction of health workers, resulting in medical unemployment in urban areas mixed with shortages in rural areas [25]. Figure 3 portrays those countries with a critical shortage of health service providers [28].

Areas with the highest global burden of disease (especially Africa and Southeast Asia) often have the fewest health care workers per capita. The growth of health systems and the demand for health workers in wealthy nations continues to draw large numbers of skilled professionals from developing countries. Shortages in rural areas of developed countries create an incentive for health workers from developing countries to relocate disproportionately compared to resident workers. In Canada, where only 9% of the physician workforce serves rural communities, the proportion of foreign physicians in rural communities without any urban influence is 30% [29]. This "brain drain" complicates any developing country's capacity to attain the WHO's recommended workforce density. Fifty-seven countries currently fall below the minimum target [30].

Total government health expenditures for health workforce average just over 40%. Only 11% of African Union Member States (6/53) have met their pledge to allocate 15% of their budget to health. War and other causes of extreme social disruption exacerbate this limited support. For example, the total number of physicians in pre-conflict Liberia was 237, dwindling to 23 after its two civil wars (1989-96, 1999-2003) [30].

These trends profoundly affect the development of an adequate global PR workforce. Many governments have been slow to acknowledge unmet needs arising during the epidemiologic shift from infectious diseases to chronic conditions. Policy must focus on the development and funding of developing nations' health workforce plans. Specifically, developing nations should be encouraged to support a long term vision that promotes coordination and training opportunities for workers at a variety of skill levels. Aside from governmental support, international donors will need to commit their resources to achieve lasting change. For the PR community, international backing substantively constitutes donated time, academic knowledge, open access journals, telemedicine capacity, minimal registration and travel costs for educational meetings, and mentoring of identified workforce resources in developing countries, many of whom are employed as civil servants [26].

Until sufficient numbers of PRs or care extenders are available, an interim policy approach suggested is "rheumatology without borders" [31]. This strategy involves structured undergraduate MSK education, current basic and clinical research training for practitioners, and petitioning of both non-governmental and national government organizations to support developing regions' providers. In part, this will require rheumatology leaders to travel to developing nations to participate in conferences, allowing participant nations' attendees affordable costs.

The PRs of Western, developed countries share an ethical imperative to provide training, appropriate resources and professional development for those health workers in developing countries. It is critical to reward these workers for their efforts, which will in turn motivate them while they mature into their roles. Of a variety of methods, PRs can contribute meaningfully by submitting their scholarly activity to open access journals. Journal participation at regional levels may also foster provider interaction with a scholarly community and the exchange of ideas. On-line courses can be translated into many languages, providing the theoretical basis of PR to health workers. Designated international centers of excellence can provide practical training for abbreviated intervals. This approach promotes sufficient training for health workers who may possess different skill mixes than physicians to become functional PR extenders in their respective regions or countries. Regulatory reform may allow realignment of clinical responsibilities through existing certifying organizations.

Infrastructure development will remain an extraordinary challenge. Developing nations (e.g., many in Africa and Asia) may benefit from strategic alignment of their economic resources akin to the process that produced the European Union. Neither profit-based health care financing nor the privately-financed charities of non-governmental organizations are likely to result in long-term, sustainable health care systems in developing countries. When governments align with their populations' needs, their countries' rheumatic disease-afflicted children will begin to receive the vital help they need.

## Summary of Policy Recommendations

While the primary focus in pediatric global health is necessarily on survival, the emerging role of chronic conditions including MSK diseases merits far-sighted development of sufficient workforce. A specific initiative by the Bone and Joint Decade addresses the urgent priority of preventing prevalent worldwide childhood mortality and morbidity from unintentional accidents [32]. Working with the WHO and the United Nations, the Bone and Joint Decade launched the "WHO Decade of Action for Road Safety 2011-2020" in May 2011. There is a need to develop pilot initiatives to address health care needs globally for children with rheumatic diseases. Internationally, potential policy solutions include:

1. Structured undergraduate MSK medicine education

2. PR faculty outreach from developed to developing countries

3. Escalation of the physician extender workforce PR training to allow the provision of limited care rather than no care

4. Regulatory reform of certifying organizations to accommodate and support these mid-level providers

5. Setting achievable health worker density targets that can be reasonably achieved within developing countries' GDP and health expenditure limits

6. Needs-based deployment of health service providers in developing countries which matches workforce distribution to accurate national epidemiologic prevalence data

7. Telemedicine programs to allow consultation and care coordination, requiring selected infrastructure development in the recipient country

8. Dispensation for providers from developing countries through multiple resources, including mentoring, medical conference costs, access to academic journals, clinical research training, online educational courses, and donated time from developed nations' PR workforce to provide these training opportunities

9. Practical, on-site training of identified providers at international centers of excellence, matching these PR extenders' anticipated practice capabilities and limitations to their developing country's health care delivery system.

## Conclusions

Expansion of the PR workforce is a strategic imperative to alleviate the persistent problems of constrained access to care. Current global demand can only be projected based upon developed nations' prevalence estimates of childhood rheumatic disease. The emergence of chronic conditions in the developing world will continue to intensify workforce demand despite limited or static population growth. The projected workforce supply needs to increase by nearly nine-fold to generate sufficient provider density. The future use of telemedicine, online PR training programs and resources represents a substantial opportunity for health care workers in developing countries. The expansion of worldwide PR workforce will require innovative approaches and tenacious advocacy with developing countries' governmental health ministries.

## Abbreviations

ACR: American College of Rheumatology; BSPAR: British Society for Paediatric and Adolescent Rheumatology; GALS: Gait, Arms, Legs, Spine; HIV/AIDS: human immunodeficiency virus/acquired immunodeficiency syndrome; ME: Middle East; MSK: musculoskeletal; OECD: Organisation for Economic Co-operation and Development; pGALS: pediatric Gait, Arms, Legs, Spine; PR: pediatric rheumatology/rheumatologist; PRES: Pediatric Rheumatology European Society; UK: United Kingdom; US: United States; WHO: World Health Organization

## Competing interests

Dr. Henrickson is a current member of the American College of Rheumatology (ACR) Committee on Government Affairs. He has no competing financial interests to disclose. The content of this article does not reflect any official position or policy of the ACR.

## Authors' contributions

MH solely contributed all aspects of this article.
